# Retraction: Emodin Inhibits Colon Cancer Cell Invasion and Migration by Suppressing Epithelial-Mesenchymal Transition via the Wnt/β-Catenin Pathway

**DOI:** 10.32604/or.2024.055032

**Published:** 2024-07-17

**Authors:** Oncology Research Editorial Office

The published article titled “Emodin Inhibits Colon Cancer Cell Invasion and Migration by Suppressing Epithelial-Mesenchymal Transition via the Wnt/β-Catenin Pathway” has been retracted from *Oncology Research*, Vol. 27, No. 2, 2019, pp. 193–202.

DOI: 10.3727/096504018X15150662230295

URL: https://www.techscience.com/or/v27n2/48644

Following the publication, concerns have been raised about a number of figures in this article. The transwell invasion assays shown in Fig. 2A were strikingly similar to data appearing in different form in other articles written by different authors at different research institutes that had already been published.

The authors were contacted and invited to comment on the concerns raised and to provide the original, unmodified figures, but did not respond. The Editors-in-Chief therefore no longer have confidence in the integrity of the data in this article and decided to retract this article.

All authors have not responded to correspondence about this retraction. As a responsible publisher, we hold the reliability and integrity of our published content in high regard. We deeply regret any inconvenience caused by this situation to our readers and all concerned parties.

